# A Serious Videogame as an Additional Therapy Tool for Training Emotional Regulation and Impulsivity Control in Severe Gambling Disorder

**DOI:** 10.3389/fpsyg.2015.01721

**Published:** 2015-11-12

**Authors:** Salomé Tárrega, Laia Castro-Carreras, Fernando Fernández-Aranda, Roser Granero, Cristina Giner-Bartolomé, Neus Aymamí, Mónica Gómez-Peña, Juan J. Santamaría, Laura Forcano, Trevor Steward, José M. Menchón, Susana Jiménez-Murcia

**Affiliations:** ^1^Department of Psychobiology and Methodology of Health Science, Universitat Autònoma de BarcelonaBarcelona, Spain; ^2^Faculty of Health and Life Sciences, Universitat Pompeu Fabra de BarcelonaBarcelona, Spain; ^3^Pathological Gambling Unit, Department of Psychiatry, Bellvitge University Hospital-IDIBELLBarcelona, Spain; ^4^Ciber Fisiopatologia Obesidad y Nutrición, Instituto Salud Carlos IIIBarcelona, Spain; ^5^Department of Clinical Sciences, School of Medicine, University of BarcelonaBarcelona, Spain; ^6^CIBERSAM - CIBER Salud Mental, Instituto Salud Carlos IIIBarcelona, Spain

**Keywords:** gambling disorder, video game therapy, impulsivity, emotion regulation, anger

## Abstract

**Background:** Gambling disorder (GD) is characterized by a significant lack of self-control and is associated with impulsivity-related personality traits. It is also linked to deficits in emotional regulation and frequently co-occurs with anxiety and depression symptoms. There is also evidence that emotional dysregulation may play a mediatory role between GD and psychopathological symptomatology. Few studies have reported the outcomes of psychological interventions that specifically address these underlying processes.

**Objectives:** To assess the utility of the Playmancer platform, a serious video game, as an additional therapy tool in a CBT intervention for GD, and to estimate pre-post changes in measures of impulsivity, anger expression and psychopathological symptomatology.

**Method:** The sample comprised a single group of 16 male treatment-seeking individuals with severe GD diagnosis. Therapy intervention consisted of 16 group weekly CBT sessions and, concurrently, 10 additional weekly sessions of a serious video game. Pre-post treatment scores on South Oaks Gambling Screen (SOGS), Barratt Impulsiveness Scale (BIS-11), I7 Impulsiveness Questionnaire (I7), State-Trait Anger Expression Inventory 2 (STAXI-2), Symptom Checklist-Revised (SCL-90-R), State-Trait Anxiety Inventory (STAI-S-T), and Novelty Seeking from the Temperament and Character Inventory-Revised (TCI-R) were compared.

**Results:** After the intervention, significant changes were observed in several measures of impulsivity, anger expression and other psychopathological symptoms. Dropout and relapse rates during treatment were similar to those described in the literature for CBT.

**Conclusion:** Complementing CBT interventions for GD with a specific therapy approach like a serious video game might be helpful in addressing certain underlying factors which are usually difficult to change, including impulsivity and anger expression.

## Introduction

Gambling disorder (GD) is currently considered as a behavioral addiction and is included in the chapter on Substance-Related and Addictive Disorders of the latest edition of the DSM (American Psychiatric Association, 2013). There is evidence of GD possessing similarities with substance use disorders (SUD) in terms of etiology, phenomenology, neurobiological mechanisms and response to treatment (Grant et al., 2010, 2013). It is widely reported that GD frequently co-occurs with SUD, and also with mood disorders and anxiety disorders (Desai et al., 2007; Petry and Weinstock, 2007; Jiménez-Murcia et al., 2009b, 2012). Several factors have been associated with severity in GD, such as early age of onset (Johansson et al., 2009; Jiménez-Murcia et al., 2015b), comorbidity (Pilver et al., 2013; Parhami et al., 2014), specific personality traits (e.g., high impulsivity; Alvarez-Moya et al., 2007; Ledgerwood et al., 2009; Maclaren et al., 2011; Black et al., 2014), or criminal behavior (Grant and Potenza, 2007; Ledgerwood et al., 2007; Folino and Abait, 2009; Granero et al., 2014b). A relationship between high impulsivity and emotional dysregulation has also been described in young adults (Schreiber et al., 2012). Various studies contend that personality traits related to impulsivity in GD (such as novelty seeking, lack of emotional control, and lack of planning) are positively associated with anger expression (Schwebel et al., 2006; Aymamí et al., 2014) and a lack of anger control (Truglia et al., 2006). Emotional regulation (ER) can be considered as a specific example of general self-regulation patterns. In this context, gambling appears to be a maladaptive strategy for regulating one's mood (Tice and Bratslavsky, 2000); one of the usual motivations for gambling is to try to relieve negative emotional states or to improve mood states (Shead and Hodgins, 2009; Lloyd et al., 2010). Furthermore, ER seems to play an important role in GD severity. Of the different subtypes of gamblers defined by Blaszczynski and Nower (2002), the two most severe subtypes, emotionally vulnerable and the antisocial impulsive, are characterized by significant emotional vulnerability and dysregulation. Other studies of subtypes in GD individuals have confirmed these findings in the general population as well as in clinical samples (Alvarez-Moya et al., 2010; Lobo et al., 2014; Suomi et al., 2014).

A wide variety of therapeutic approaches to GD have been described, from self-management interventions to professionally delivered treatments. Presently, interventions based on cognitive-behavioral therapy (CBT) are the most frequently applied therapy for GD (Rash and Petry, 2014). Nevertheless, CBT presents several major limitations: dropout rates above 30% (Jiménez-Murcia et al., 2007; Melville et al., 2007), relapse rates ranging from 14.5 to 18.5% following treatment (Hodgins and el-Guebaly, 2004; Ledgerwood and Petry, 2006; Jiménez-Murcia et al., 2007), and low compliance with treatment, fundamentally due to low motivation to change (Jiménez-Murcia et al., 2007; Gómez-Peña et al., 2012). Some evidence suggests that personality traits such as impulsivity in combination with deficits in self-regulation may predict dropout (Leblond et al., 2003; Alvarez-Moya et al., 2011) and other outcomes in CBT for GD (MacCallum et al., 2007; Alvarez-Moya et al., 2011). That being the case, trait- or symptom-oriented interventions may optimize response to CBT in GD patients. Various authors coincide in stressing the relevance of impulsivity, anger expression and ER in treatment programs for GD (First et al., 1997; Aymamí et al., 2014). Moreover, these underlying factors may be more difficult to modify.

Although the role of ER in the development, maintenance and treatment of psychopathology has been largely proven by the literature (Berking et al., 2008, 2013; Berking and Margraf, 2011; Sheppes et al., 2015), there is as yet no consensus on what the most suitable treatment approach may be. Several studies have assessed assorted ER therapies for different disorders such as major depression, eating disorders, personality disorders, or SUD (Ducharme et al., 2012; Price et al., 2012; Fagundo et al., 2013, 2014; Gratz et al., 2014; Radkovsky et al., 2014; Wallace et al., 2014). A wide variety of therapeutic approaches can be applied, though the most frequent are CBT, variations of it (Ducharme et al., 2012; Fagundo et al., 2013, 2014; Radkovsky et al., 2014) and dialectical behavior therapy (Geddes et al., 2013; Wallace et al., 2014).

Several new treatment perspectives in ER therapy have emerged in last decade and of particular interest are interventions using computer-based ER training or serious video-games (Hobbs and Yan, 2008; Jiménez-Murcia et al., 2009a; Kazdin and Blase, 2011; Ducharme et al., 2012; Fernández-Aranda et al., 2012). Although these innovative treatment approaches could be more cost-effective, therapeutic strategies based on the new technologies is still limited (Santamaría et al., 2011).

PlayMancer (PM) is a serious videogame specifically designed to treat impulse control disorders (Jiménez-Murcia et al., 2009a; Fernández-Aranda et al., 2012). The objective of the game is to enhance self-control and general impulsive behaviors and emotional skills via training that reduces arousal and improves decision-making and planning.

PM uses biofeedback to model physiological and emotional reactions. Some literature reviews support that biofeedback-based tools are useful for treating those psychiatric disorders in which maladaptive physiological mechanisms are a relevant maintaining factor, as biofeedback contributes to becoming aware of one's own physiology and facilitates enhancing self-regulation (Schoenberg and David, 2014). Other studies have shown that biofeedback interventions effectively address impulse control difficulties and improve attention deficits in different psychopathological disorders (Howard et al., 2013) including impulse-related disorders (Fagundo et al., 2013, 2014; Giner-Bartolomé et al., 2015). Intensive biofeedback and relaxation training have also been shown to have a positive impact on stress, anxiety, and anger indices (Pawlow et al., 2003). As a psychotherapy tool, this videogame has provided promising results in the treatment of bulimia nervosa in female patients, suggesting that combining CBT and PM could potentially improve emotional regulation and impulsivity control (Fagundo et al., 2013, 2014; Giner-Bartolomé et al., 2015).

The positive features offered by video games (e.g., intensiveness, immersive capacity, and low resistance) makes PM an ideal candidate for addressing the underlying cognitive and emotional processes that are otherwise difficult to treat (Fernández-Aranda et al., 2012). Given the important relationship between impulsivity, ER, GD severity and treatment response described above, assessing the feasibility of including PM as a complementary therapy tool in severe gamblers treatment is worthy of consideration. The aims of this pilot study were to incorporate a serious videogame as a complementary therapy tool into a CBT program in a male sample with severe GD and to evaluate its possible additional impact on impulsivity traits, anger expression, and emotional distress.

## Methods

### Participants

The sample consisted of 16 consecutive male patients diagnosed with GD who were undergoing treatment at the Pathological Gambling Unit in the Psychiatry Department of the University Hospital of Bellvitge (Barcelona, Spain) and who agreed to participate in this study. The hospital is a public hospital certified as a tertiary care center for the treatment of GD. The catchment area of the hospital covers over two million people south of the Barcelona metropolitan area.

Patients were assessed by expert and experienced clinical psychologists and psychiatrists in the field of GD and diagnosed according to DSM-IV (American Psychiatric Association, 2000) using the Stinchfield Diagnostic Questionnaire for Pathological Gambling (Stinchfield, 2003; Jiménez-Murcia et al., 2009c) and the DSM-5 diagnostic criteria (American Psychiatric Association, 2013). All participants were also screened for Internet Gaming Disorder, following the criteria proposed in Section III of the DSM-5 (American Psychiatric Association, 2013).

Exclusion criteria were primary psychiatric or neurological disorders that might interfere with game performance (psychotic disorders, bipolar disorders, major depressive disorders, and substance abuse-disorders) measured by means of the structured clinical interview for DSM IV (SCID-I; First et al., 1997), active pharmacological therapy that might influence autonomic functioning or interfere with game performance, and current or lifetime diagnosis of Internet Gaming Disorders (American Psychiatric Association, 2013).

Gambling behavior was measured with the South Oaks gambling Screen (SOGS). At baseline, 100% of patients reported playing slot machines, 86.7% lotteries, 40% bingo, and 20% casino games. The majority of patients had more than one problematic gambling behavior, as is usual in most severe cases. Other noteworthy characteristics include: 93.3% of the sample reporting going back to win back lost money, 60% claiming to have won money when in fact having lost money, 100% experiencing guilty feelings, 93.3% hiding signs of gambling, and 76.9% admitting to having arguments and fights related with this problem. Table 1 includes other clinical and sociodemographic characteristics of the initial sample.

**Table 1:** **Descriptives for the sample**.

Origin; % Spain	93.8
Education level; % primary or less	75.0
Secondary	18.8
University	6.25
Civil status; % single	46.7
Married/couple	33.3
Divorced/separated	20.0
Employment stat.; % employed	68.8
Own incomes (euros); mean (*SD*)	1205.7 (523.1)
Family incomes (euros); mean (*SD*)	1928.8 (1085.2)
Previous GD treatments; %	43.8
Present comorbid disorders; %	28.6
Previous comorbid disorders; %	57.1
Smoker (yes); %	62.5
Alcohol abuse (yes); %	21.4
Other drugs abuse (yes); %	7.14
Age (years); mean (*SD*)	34.8 (6.02)
Duration gambling probl. (years); mean (*SD*)	5.97 (5.06)
Age of onset (years); mean (*SD*)	28.8 (7.39)
SOGS-total; mean (*SD*)	11.2 (3.10)
DSM-IV-total; mean (*SD*)	8.27 (1.44)
Illegal acts; %	43.8
Maximum bets (euros); mean (*SD*)	770.0 (859.0)
Mean bets (euros); mean (*SD*)	160.0 (247.9)
Cumulated debts (euros); mean (*SD*)	7555.6 (11,555.4)

SD, standard deviation (n = 16).

## Measures

### Gambling behavior

#### South Oaks Gambling Screen (SOGS; Lesieur and Blume, 1987)

This is a self-report gambling questionnaire. The total score ranges from 0 to 20. The Spanish validation of this questionnaire showed high internal consistency (0.94) and good test–retest reliability (0.98) (Echeburúa et al., 1994). Internal consistency in our sample was excellent (0.87).

#### Stinchfield's diagnostic questionnaire for pathological gambling according to DSM-IV criteria (Stinchfield, 2003); spanish validation (Jiménez-murcia et al., 2009c)

This 19-item questionnaire measures the DSM-IV diagnostic criteria for pathological gambling. The Spanish version has shown excellent internal consistency (0.95). Cronbach's alpha in the sample was very good (0.80).

### Impulsivity, personality, and psychopathological status

#### Barratt impulsiveness scale (BIS-11; Patton et al., 1995)

The BIS-11 includes 30 items rated on a four-point Likert scale. The total score can range from 30 to 120. It comprises three subscales: cognitive impulsiveness, motor impulsiveness and non-planning impulsiveness and a total score. Cronbach's alphas in the sample for the subscales and the total score were 0.33, 0.82, 0.60, and 0.85, respectively.

#### I7 impulsiveness questionnaire (I7; Eysenck et al., 1985)

This is a 54-item self-report scale in a yes/no format that measures two dimensions of impulsivity (impulsiveness and venturesomeness) and one dimension of empathy. Cronbach's alpha reliability in the sample for these three dimensions was 0.87, 0.62, and 0.58, respectively.

#### State-trait anxiety inventory (STAI-S-T; Spielberger et al., 1970)

State-Trait Anxiety Inventory (STAI-S-T; Spielberger et al., 1970) is a self-report questionnaire which evaluates “state anxiety” and “trait anxiety” and includes 40-item with a 1–4 response scale. The Spanish adaptation (Spielberger et al., 1982) achieved good reliability indices in the psychometric studies carried out in Spanish population (Guillén-Riquelme and Buela-Casal, 2011). Cronbach's alpha reliability in sample was excellent (0.89 for Trait dimension and 0.90 for State dimension).

#### State-trait anger expression inventory 2 (STAXI-2; Spielberger, 1999)

This self-report instrument examines the experience and expression of anger. The Spanish version comprises 49 items (Miguel-Tobal et al., 2001) structured in six scales. Items are rated on four-point Likert scales. Only the State Anger, Trait Anger and Anger Expression Index scales were used. Cronbach's alpha reliability for these three scales in the sample was 0.99, 0.95, and 0.88, respectively.

#### Symptom check list–90 items-revised (SCL-90-R; Derogatis, 1997)

Symptom Check List–90 items-Revised (SCL-90-R; Derogatis, 1997), this questionnaire includes 90 items with a five-point scale format, evaluates nine primary symptom dimensions and includes three global indices. In this study, only depression, anxiety, and hostility subscales and the three global indices global severity index (GSI), positive symptom distress index (PSDI) and positive symptom total (PST) were used. The Spanish validation obtained adequate internal consistency and an acceptable mean internal consistency (Martínez-Azumendi et al., 2001). Internal consistency in the sample ranged between very good to excellent (0.76 and 0.98).

#### Temperament and character inventory-revised (TCI-R)

This is a 240-item questionnaire with a five-point Likert scale format used to measure four temperament and three character dimensions of personality. Spanish adaptation (Gutiérrez-Zotes et al., 2004) has shown adequate reliability of the different personality dimensions. In this study, we only analyzed novelty seeking dimension scores. Internal consistency for this scale in sample was good 0.75.

#### Other sociodemographic and clinical variables

Additional demographic, clinical, and social/family variables related to gambling were measured using a semi-structured face-to-face clinical interview described elsewhere (Jiménez-Murcia et al., 2007). Throughout the treatment period, attendance, control of spending and gambling behavior, compliance with treatment (subjectively rated by the therapist as good, fair, or poor) were recorded on an observation sheet, as well as the occurrence of relapse, desire or urge to gamble, avoidance of risk situations and instructions of tasks to be completed for the following session. The observation sheet was completed during the treatment session by both the therapist and co-therapist. At the end of the session, the records were compared in order to judge the level of inter-rater agreement.

## Procedure

Expert and experienced psychologists and psychiatrists conducted the first two face-to-face clinical interviews. In addition to a comprehensive clinical and psychological evaluation including the use of the instruments mentioned above, demographic data were recorded at the beginning of therapy. Patients were also assessed during the last therapy appointment (in the 16th session).

The study was carried out in accordance with the latest version of the Declaration of Helsinki, and was approved by the University Hospital of Bellvitge' Clinical Research Ethics Committee (ref. PR098/09). Written and signed informed consent was obtained from all participants.

### Cognitive-behavioral treatment

Patients were assigned to an outpatient CBT group receiving 16 weekly sessions lasting 90 min each. Groups were led by an experienced clinical psychologist aided by another clinical psychologist acting as a co-therapist. The goal of the treatment was to train patients to implement CBT strategies in order to achieve full, definitive abstinence from all types of gambling. As previously described (Jiménez-Murcia et al., 2015a), the general topics addressed included: psychoeducation for GD, stimulus control, response prevention, cognitive restructuring focused on illusions of control over gambling and magical thinking, reinforcement and self-reinforcement, skills training, and relapse prevention techniques. This treatment program and the accompanying materials have already been published in Spain (Jiménez-Murcia et al., 2006). The short- and medium-term effectiveness of this group CBT approach and the predictors of therapy outcome have been demonstrated in previous research (Jiménez-Murcia et al., 2007, 2012, 2015a).

### The video game intervention

A detailed description of the *Playmancer* video game (VG) is available in Fernández-Aranda et al. (2012) and Jiménez-Murcia et al. (2009a).

**Figure 1:**
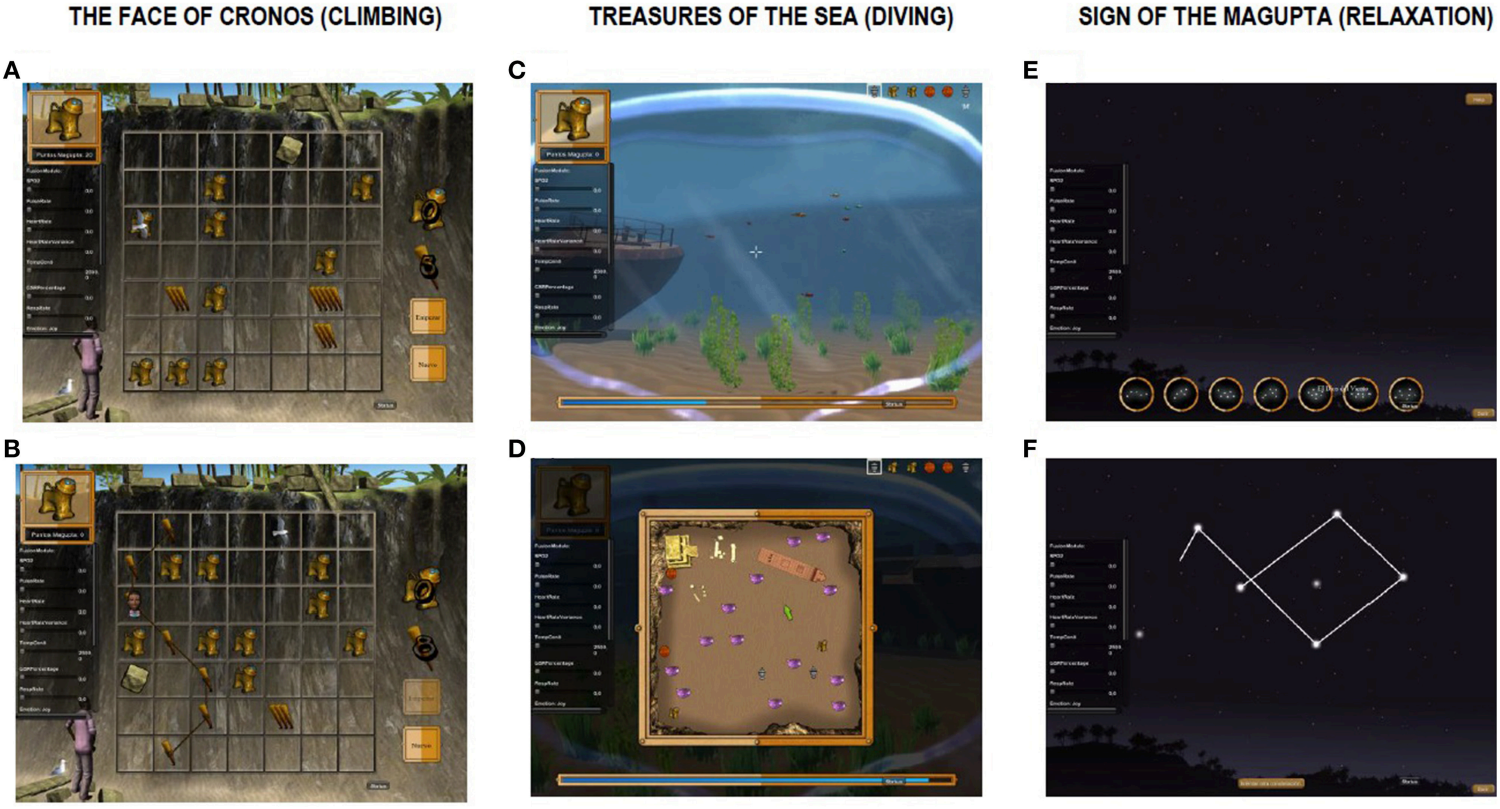
**Description and scenarios examples of the Playmancer minigames**. The player has to discover the most efficient way to climb while picking up as many treasures they can, as well as needed materials **(A)**; Obstacles such as rocks or birds appear depending on the player's arousal level, which is based on biofeedback **(B)**. The player has to dive into the sea to gather as many treasures as they can and be provided oxygen by fish distributed throughout the scene **(C)**; Simultaneously, the player must plan the most efficient route in order to preserve their oxygen supply, which is shown at the bottom of the screen **(D)**. High arousal makes the task more difficult (e.g., it is harder to catch oxygen-providing fish and the player's oxygen supply runs out faster). The player has to control their breathing in order to connect a constellation of stars of varying difficulty **(E)**; Slow, deep breathing allows the connections between stars to form **(F)**.

The overall goal of this VG is to improve problem solving, planning and self-control skills as well as control over general impulsive behaviors and relaxation skills. During a *Playmancer VG whole* session, the physiological reactivity and emotional state of the patient are continuously being monitored and is used to modify the difficulty level of the video game. The higher undesired emotional and/or physiological reactions, the greater the difficulty to reach the end of the VG. Likewise, the more relaxed and self-controlled reactions are, the easier it is to reach the VG goals. Moreover, *Playmancer VG* leads the patient to a relaxing environement every time undesired emotional and/or physiological reactions are detected. *Playmancer VG* includes three mini-games of increasing difficulty which are described in Figure 1. Table 2 shows the psychopathological targets and therapy goals of Playmancer VG and how each mini game is expected to allow each player to achieve them.

**Table 2:** **Correspondence between psychopathological target and therapy goals for which mini games were designed**.

**Psychopathological target**	**Therapy goals**	**Related mini game**		
Impulsive behaviors	Enhance planning skills	The face of Cronos (climbing)	Treasures of the sea (diving)	
Lack of boredom management	Learning to delay impulsive responses			
Low tolerance to cope with adversities	Improve tolerance to cope with adversities, handle to cope with adversities and consequent disappointment			
Lack of stress management	Learning stress management			
Strong negative emotional expression in front of minimal stimuli	Emotional self-regulation, reacting in a more controlled way, from emotional and physiological point of view			
High physiological reactivity in front of stress	Increase physiological and emotional awareness and self-control	The face of Cronos (climbing)	Treasures of the sea (diving)	Sign of the Magupta (relaxation)
	Learning and training relaxation and breathing techniques			
	Self-soothing and self-regulation skills (distracting, self-soothing, imagery, relaxation, etc.)			

**Figure 2:**
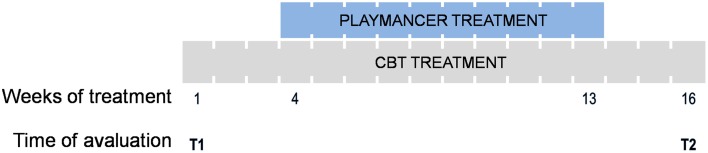
**Schematic diagram of treatment: cognitive behavior therapy (CBT) and Playmancer duration; and the time points evaluated T1 (pre-treatment) and T2 (post-treatment)**.

**Figure 3:**
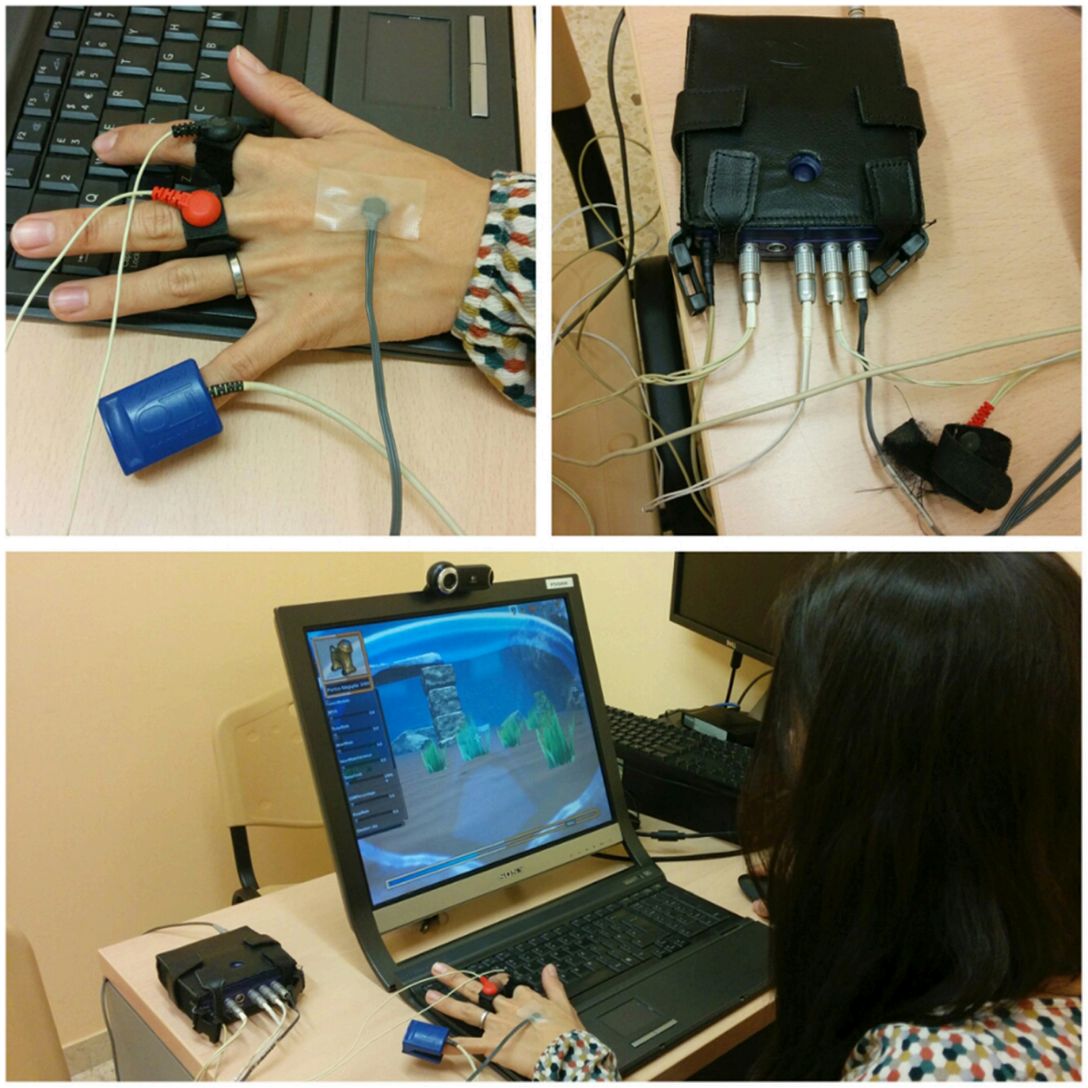
**Recording physiological activity during the Playmancer VG session**.

A total of 10 sessions were carried out once a week (Figure 2), on the day of patients' usual CBT therapy, and consisted of 20 min exposure to the above-mentioned VG. Relaxing music was played for 3 min before and after each VG session. Biosensor assessment measures were taken before, during and after the session. Figure 3 depicts the recording of physiological activity during the *Playmancer VG* session.

## Statistical analysis

Analyses were carried out with SPSS20 for Windows. First, *t*-tests for paired samples explored pre-post changes for BIS-11, I7, STAI S-T, STAXI S-T, SCL-90-R Hostility, SCL-90-R Depression, SCL-90-R anxiety, and TCI Novelty Seeking scores. The effect size was assessed by the Cohen's d coefficient (effect sizes were considered medium for |*d*| > 0.50 and good for |*d*| > 0.80). Second, the Kaplan–Meier function estimated the cumulate survival function for the presence of relapses during the therapy. The Kaplan–Meier function was included in the survival techniques for censored data, a group of statistical procedures used to describe the time duration until the appearance of an event. In this study, the Kaplan–Meier function estimated the fraction of patients “surviving without relapses” during the therapy. Drop-outs were defined as missing group sessions on three or more consecutive occasions without notifying the therapist. Relapses were defined as the presence of gambling episodes during the treatment. The therapy session in which the first relapse or the drop-out was recorded was the measure of survival time.

## Results

### Changes in SOGS total score, impulsivity, anger, and emotional distress at the end of the therapy

Post-treatment data were analyzed for the *n* = 13 patients who completed therapy. As a whole, post-therapy mean scores were lower than baseline scores (Table 3). Baseline scores were high on some impulsivity-related measures: TCI-Novelty Seeking scores, BIS-Cognitive Impulsivity, BIS-Unplanned, and I7 Impulsivity.

**Table 3:** **Comparison for the psychometrics scores**.

	**Means (*****SD*****)**				**Pre vs. Post**					
	**Pre**		**Post**		***MD***	***SE***	***p***	***IC* 95%**		**|*d*|**
SOGS	11.3	(2.74)	7.58	(5.96)	−**3.75**	5.82	**0.047**	−7.45	0.05	**0.81**^††^
BIS: Cognitive impulsiveness	16.4	(4.60)	13.9	(2.94)	−**2.50**	1.12	**0.048**	−4.98	−0.02	**0.65**^†^
BIS: Motor impulsiveness	17.8	(7.52)	15.3	(4.62)	−2.42	1.80	0.206	−6.37	1.54	0.39
BIS: Unplanned impulsiveness	24.9	(6.14)	19.1	(4.50)	−**5.83**	2.22	**0.023**	−10.7	−0.95	**1.08**^††^
BIS: Total	59.3	(15.6)	48.3	(8.87)	−**10.9**	4.33	**0.028**	−20.5	−1.38	**0.86**^††^
I7: Impulsivity	10.4	(4.76)	7.50	(4.30)	−**2.92**	1.24	**0.038**	−5.65	−0.19	**0.64**^†^
I7: Adventure	9.00	(3.36)	9.08	(3.87)	0.08	0.47	0.862	−0.95	1.11	0.02
I7: Empathy	12.1	(2.81)	12.0	(2.45)	−0.08	0.71	0.909	−1.65	1.48	0.03
STAI: State	20.3	(9.75)	19.3	(10.6)	−1.00	3.06	0.750	−7.72	5.72	0.10
STAI: Trait	24.2	(7.96)	18.5	(8.19)	−**5.64**	1.32	**0.002**	−8.58	−2.69	**0.70**^†^
STAXI: State	17.6	(4.08)	16.5	(6.07)	−1.08	1.26	0.410	−3.87	1.70	0.21
STAXI: Trait	19.3	(6.10)	17.1	(5.63)	−2.25	0.99	0.045	−4.44	−0.06	0.38
STAXI: Anger expression	30.3	(13.0)	24.4	(8.80)	−**5.92**	3.27	0.098	−13.1	1.28	**0.53**^†^
SCL: Hostility	1.17	(0.72)	0.37	(0.40)	−**0.80**	0.21	**0.003**	−1.26	−0.34	**1.38**^††^
SCL: Depression	1.32	(0.76)	0.61	(0.65)	−**0.71**	0.24	**0.012**	−1.23	−0.19	**1.01**^††^
SCL: Anxiety	1.10	(0.63)	0.47	(0.48)	−**0.63**	0.19	**0.006**	−1.04	−0.22	**1.13**^††^
SCL: GSI	1.04	(0.67)	0.50	(0.50)	−**0.53**	0.21	**0.027**	−1.00	−0.07	**0.90**^††^
SCL: PST	47.4	(22.3)	26.9	(20.4)	−**20.5**	8.43	**0.032**	−38.8	−2.09	**0.96**^††^
SCL: PSDI	1.74	(0.70)	1.32	(0.55)	−**0.42**	0.20	0.055	−0.85	0.01	**0.67**^†^
TCI: Novelty seeking	119.7	(9.67)	110.8	(12.9)	−**8.85**	3.52	**0.027**	−16.5	−1.19	**0.77**^†^

SD, standard deviation; Bold, Significant comparison (0.05 level); |d|, Cohen's-d coefficient;

† Moderate(|d| > 0.50) to

†† high (|d| > 0.80);

effect size (n = 13).

The changes were significant and their size effects were moderate to high (ranging from 0.64 to 1.08) on SOGS total scores, all BIS scores except for BIS Motor Impulsiveness, and I7 Impulsivity. All the SCL-90-R scale scores evaluated experimented significant pre-post changes with a high effect size (from 0.96 to 1.38) except for the SCL PSDI mean scores (on which the change was not significant and the effect size was moderate).

STAI-Trait, STAXI anger expression and TCI Novelty Seeking mean scores also achieved notable post-therapy changes with a moderate effect size (from 0.53 to 0.77), although the difference for STAXI anger expression was not significant.

### Dropouts and relapses during treatment

Two participants (12.5%) dropped out during treatment. One drop-out was recorded at the end of the first month (with a cumulative survival probability after 4 weeks of 93.8%) and the other was recorded at week 13 (with a cumulative survival probability at session 13 of 87.5%).

**Figure 4:**
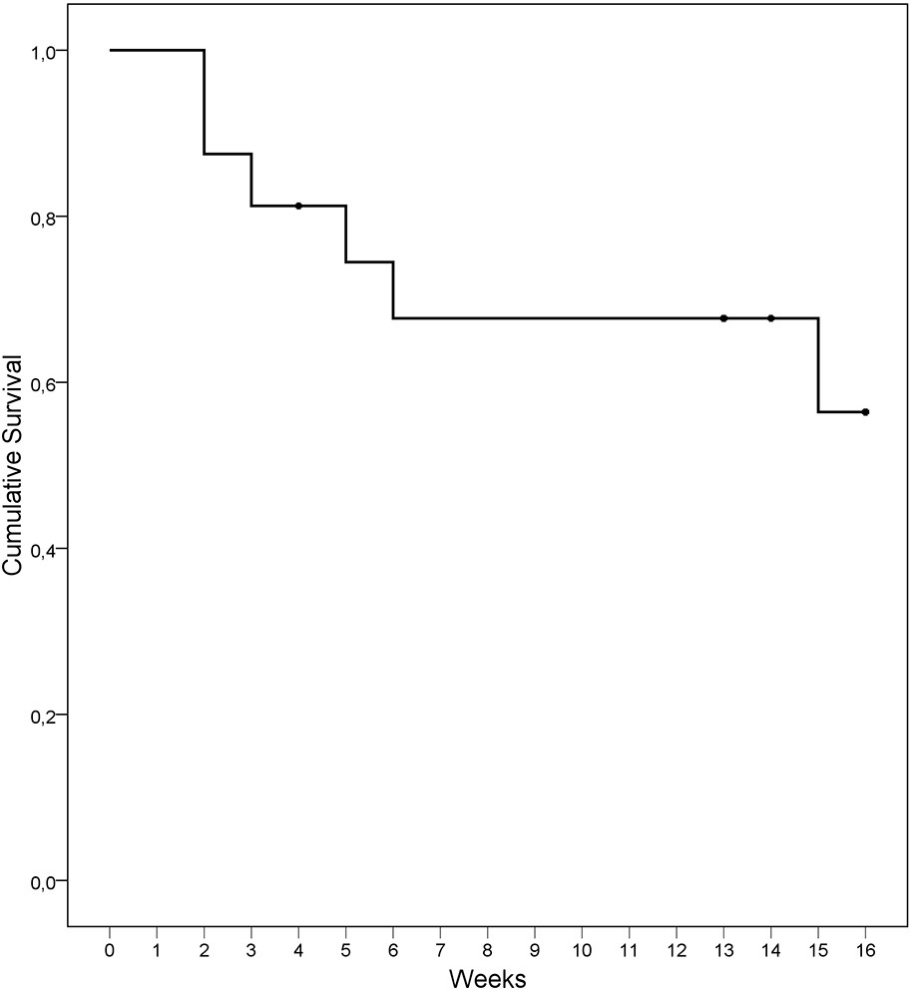
**Kaplan–Meier curve for the cumulative survival of relapses during treatment (*n* = 16)**.

Figure 4 shows the survival function for the occurrence of relapses during the treatment. Six patients (37.5%) reported at least one relapse during the therapy sessions. Three patients (18.75% of the sample) reported the first relapse during the first month (the cumulative survival probability after 4 weeks was 81.25%). Two additional patients (12.5%) relapsed between weeks 5 and 6 (with a cumulative survival probability at session 6 of 67.71%). Only one patient (6.25%) reported his first relapse episode at the end of the treatment (week 15).

## Discussion

The aims of this pilot study were to evaluate the feasibility of using Playmancer VG as an additional therapy tool in a CBT intervention for GD, and to assess treatment dropout and relapse rates, and pre-post treatment changes in impulsivity, anger expression and emotional distress.

As reported above, our sample comprised male slot-machine gamblers, with ages ranging from 24 to 46 (mean 34.8 years old) and most had secondary studies or lower, were either single or divorced, and employed. In agreement with the literature, high rates of current and previous comorbid disorders and SUD were observed in our sample (Ledgerwood et al., 2007; Jiménez-Murcia et al., 2009b; Lorains et al., 2011). Comorbidity in problematic and disordered gamblers has been associated with more gambling problems and higher severity of the associated consequences (Bischof et al., 2014; Black et al., 2014). The DSM-IV total criteria recorded indicated that the degree of gambling severity in our sample was moderate to high. One of the most notable severity indicators was the high proportion of patients reporting illegal acts (43.8%) compared with the rates in previous literature (14–30%) (Ledgerwood et al., 2007; Granero et al., 2014a) in fact, the association between criminal behavior and GD severity is well-established in several studies (Grant and Potenza, 2007; Ledgerwood et al., 2007; Folino and Abait, 2009; Granero et al., 2014b). As expected, high levels of impulsivity were observed at baseline (Alvarez-Moya et al., 2007; MacCallum et al., 2007; Ledgerwood et al., 2009; Maclaren et al., 2011; Petry et al., 2014), as in other studies which included GD patients who have committed illegal acts (Jiménez-Murcia et al., 2013; Granero et al., 2014b).

In general, the sample characteristics were also quite consistent with the most disturbed subtype of gamblers defined by Blaszczynski and Nower (2002)—the antisocial impulsive subtype—and with Subtype I in the study by Granero et al. (2014a) of GD patients who presented concurrent illegal behaviors. These gamblers are likely to present high levels of psychopathology and to show significant emotional vulnerability, probably involving greater emotional dysregulation than the emotionally vulnerable typology. In comparison with the other subtypes, the antisocial impulsive subtype is characterized by high levels of impulsive traits; these patients appear to be more likely to engage in criminal acts, to have drug or alcohol problems, and to exhibit antisocial traits.

Several studies have linked GD with anger and with aggressive behaviors and violence (Cunningham-Williams et al., 2009; Dowling et al., 2014). There is also some evidence of a relationship between high novelty seeking, hostility, anger and severity of GD in treatment-seeking individuals (Aymamí et al., 2014). Some authors found that people with GD reported a lower level of awareness or insight into their emotions than healthy controls (Williams et al., 2012).

With regard to the capacity of the therapy to achieve change, notable pre-post changes were observed in several of the mean scores evaluated: BIS cognitive Impulsiveness, BIS Unplanned Impulsiveness, I7 Impulsivity, Psychopathology distress evaluated with SCL-90-R scales, STAI-Trait, STAXI anger expression and TCI Novelty Seeking. Our findings support the evidence that CBT is an effective therapy for GD and for improving emotional distress levels (Petry et al., 2006; Cowlishaw et al., 2012; Rash and Petry, 2014). However, the previous literature on ER in GD is scarce, particularly with regard to the assessment of interventions for training emotion regulation and self-control.

As for treatment outcomes, previous studies have reported dropout rates between 14 and 50% (Jiménez-Murcia et al., 2007; Melville et al., 2007; Pasche et al., 2013). Our dropout results were within this range. However, it is important to note that the patients included in our study had moderate to severe GD, with high levels of impulsivity, sensation seeking, anger, hostility, and emotional distress. Almost half of the sample had committed illegal acts and had been charged with a felony, such as violent robbery and theft of large sums of money from the bank where the patient had worked. In both cases, these criminal acts were the result of their gambling problems, with these cases not having presented a history of crimes or offenses prior to the development of the disorder. Following Folino and Abait (2009), criminal behavior and illegal acts are indicators of gambling disorder severity and require specific therapeutic strategies. Overall, these characteristics have been associated with poor prognosis and poor response to treatment (Blaszczynski and Nower, 2002; Alvarez-Moya et al., 2010; Aragay et al., 2015; Jiménez-Murcia et al., 2015a). However, dropouts reported in the current study were lower than could normally be expected. Some studies have shown that using video games could enhance motivation and treatment adherence (Kato et al., 2008; Coyle et al., 2009). In fact, the addition of interventions aimed at improving gamblers' motivation to CBT are useful for reducing dropout and increasing treatment adherence, as well as for facilitating therapeutic success (Melville et al., 2007). Previous studies carried out with *Playmancer* VG in eating disorder and gambling disorder patients suggest that it has high acceptability and that playing is an enjoyable (usability about 86%) and positive experience for such patients (Fernández-Aranda et al., 2012; Fagundo et al., 2013; Giner-Bartolomé et al., 2015). PM, as an adventurous VG, could be contemplated by patients as a recreational activity since it does not directly address core gambling symptoms and for this reason, receive lower resistance than other therapy approaches. Therefore, PM might help in the treatment adherence and that could possibly be explained for PM's capability to maintain patients' motivation, as has been argued in other studies (Fagundo et al., 2013).

Even though 37.5% of patients presented relapses during treatment, almost all relapses were recorded during the first 6 weeks. Nearly all the patients who had remained abstinent until that point finished the treatment without relapsing. Although some studies report relapse rates between 14.5 and 18.5% during treatment (Aragay et al., 2015), others describe higher percentages (especially those that applied a strict definition of relapse, as the present study does—any gambling episode after beginning the attempt to abstain; Gómez-Peña et al., 2012; Jimenez-Murcia et al., 2012). This definition of relapse is similar to the one considered by Hodgins and el-Guebaly (2004). Moreover, in general, the severity of the disorder, high impulsivity and sensation seeking levels and comorbidity are often poor predictors of treatment response (Jiménez-Murcia et al., 2015a), coinciding with the clinical profile of patients in our sample.

This is, to the best of our knowledge, the first time that a serious videogame therapy has been used as a therapeutic intervention in GD patients. Taking into account the sample characteristics (moderate-high severity of GD, high impulsivity, the significant rates of comorbidity and SUD, and the high rates of illegal acts) poorer clinical outcomes might be expected, since various studies have indicated that impulsivity is a significant factor in treatment dropout (Leblond et al., 2003; Alvarez-Moya et al., 2011; Jiménez-Murcia et al., 2015a), and that treatment outcomes in individuals with SUD and concurrent antisocial traits have been reported to be poorer than in individuals without such traits (Compton et al., 2003). Nevertheless, CBT complemented with the PM intervention obtained similar outcomes to CBT for treatment-seeking gamblers reported by previous studies (Jiménez-Murcia et al., 2007, 2015a). In this study though, a positive impact on self-reported scores for impulsivity traits, hostility, and anger expression was found and our findings suggest that complementing CBT with PM could have additional positive effects on self-control and ER. While this may be the first study to a describe the results of treatment base on a serious video game, as previously mentioned, more and more healthy professionals are showing interest in the potential of these interventions (Santamaría et al., 2011; Claes et al., 2012; Fernández-Aranda et al., 2012; Botella et al., 2013). The application of these novel interventions for somatic and psychological disorders as a complement to- or enhancer of conventional therapy is becoming of general interest (Barnett et al., 2011). Videogame-based interventions could prove to be successful as an educational tool in learning and training skills, especially in they are designed to target concrete problems or specific skills (Griffiths, 2004; De Freitas and Griffiths, 2007).

The results of this study should be interpreted bearing in mind its preliminary nature and the following limitations. Firstly, the generalization of the results described is quite limited. The sample was small, and consisted of seeking-treatment male patients who were mainly slot-machine gamblers. Moreover, the patients who decided to enroll in this study presented a significant level of severity. Some evidence suggests that severe patients may be more motivated to change their gambling behavior, because the severity might make them more aware of the negative consequences of their disorder (Gómez-Peña et al., 2012). Secondly, it would have been desirable to be able to include a control group with similar features. Thirdly, main results were based on self-reported outcomes therefore; results could have been interfered with by a possible expectation bias. To conclude, it seems important to find new therapeutic approaches to treat GD, especially in cases with more severe symptoms, high levels of impulsivity, and impaired emotion regulation. The sample described in this study also presented high rates of criminal behavior associated with gambling problems. The encouraging results show that new technologies can be useful therapeutic strategies that merit further exploration.

The clinical relevance of this study lies in the finding that severe patients with GD may respond satisfactorily to CBT with the incorporation of new therapeutic alternatives. New technologies, and more specifically serious games, are innovative tools which are highly motivating for most users and may allow for treatment of the underlying psychological aspects involved in certain disorders, such as impulsivity, self-control problems, or deficits in ER.

### Conflict of interest statement

The authors declare that the research was conducted in the absence of any commercial or financial relationships that could be construed as a potential conflict of interest.
